# Transcriptome analysis and RT-qPCR validation of mitophagy-related key genes in the progression of diabetic retinopathy

**DOI:** 10.3389/fendo.2025.1733368

**Published:** 2026-01-12

**Authors:** Yifan Zhang, Liangjie Niu, Huika Xia, Jianmin Wang

**Affiliations:** 1 Ophthalmology Department, Hebei General Hospital, Shijiazhuang, China; 2 Ophthalmology Department, The Second Hospital of Hebei Medical University, Shijiazhuang, China

**Keywords:** bioinformatics analysis, diabetic retinopathy, mitophagy-related genes, RPS21, SLC1A5

## Abstract

**Background:**

Diabetic retinopathy (DR), a prevalent microvascular complication of diabetes mellitus (DM), likely involves mitophagy in its progression. However, the exact mechanisms remain poorly understood.

**Methods:**

This study analyzed DR datasets GSE189005 and GSE221521 from the Gene Expression Omnibus (GEO) database. Differentially expressed genes (DEGs 1) between patients with DR and controls were identified from GSE189005. Simultaneously, mitophagy-related genes (MRGs) were analyzed to determine DEGs 2. The datasets were integrated to obtain differentially expressed MRGs. A machine learning-based approach was used to identify key candidate genes, followed by expression validation. Additionally, a nomogram was constructed for DR risk prediction; correlation analysis was performed between key genes and immune cells; RT-qPCR analysis was conducted to verify gene expression.

**Results:**

Integration of datasets revealed 13 differentially expressed MRGs. Five key candidate genes were identified via machine learning, and expression validation confirmed the differential expression of SLC1A5 and RPS21 in DR. The nomogram incorporating these two genes showed high predictive accuracy for DR risk. SLC1A5 was strongly positively correlated with CD56 bright NK cells (r = 0.82) and negatively correlated with CD56 dim NK cells (r = -0.80). RPS21 exhibited the strongest positive correlation with CD56 dim NK cells (r = 0.77) and the strongest negative correlation with CD56 bright NK cells (r = -0.75). RT-qPCR analysis indicated significant upregulation of SLC1A5 and downregulation of RPS21 in DR samples.

**Conclusions:**

This study suggests that SLC1A5 and RPS21 are involved in DR progression, offering potential therapeutic targets. However, further experimental validation is necessary to confirm their functional roles and clinical relevance.

## 1 Introduction

Diabetic retinopathy (DR) is a prevalent and distinct complication of diabetes mellitus (DM), characterized by retinal neovascularization and macular edema due to fluid accumulation in the retina (1). DR has emerged as a leading global cause of visual impairment and blindness, significantly affecting patients’ quality of life and imposing a considerable societal healthcare burden (2). The incidence of DR correlates directly with the prevalence of DM, and with the rising rates of DM and increasing life expectancy, the prevalence of DR is expected to continue to rise (3). Current treatments for DR primarily address microvascular complications through methods such as intravitreal drug injections, retinal laser photocoagulation, and vitrectomy. However, these interventions often have side effects, including gradual loss of vision and visual field (4). Additionally, most patients with DR experience irreversible visual function damage by the time surgery is required, with no recovery following the procedure (5). Therefore, understanding the molecular mechanisms underlying DR onset and progression, as well as identifying strategies to prevent its progression, has become a critical research focus.

Mitochondria, double-membraned organelles found in most eukaryotic cells, are responsible for producing adenosine triphosphate (ATP), the primary cellular energy source (6). Mitochondria are regulated by dynamic processes such as biosynthesis, fission and fusion, autophagy, and transport, which facilitate the generation of new mitochondria, maintain mitochondrial morphology, eliminate damaged or dysfunctional mitochondria, and preserve mitochondrial homeostasis (7). Mitophagy, a selective form of autophagy distinct from other cellular autophagic processes, involves the degradation of dysfunctional mitochondria *via* the autophagolysosomal pathway, preventing further cellular damage caused by these defective mitochondria (8). Disruption of mitophagy impairs mitochondrial function, leading to the accumulation of reactive oxygen species (ROS) in damaged mitochondria, contributing to cellular degeneration and tissue damage (9). Abnormal mitophagy has been implicated in various diseases, including liver, cardiovascular, and neurodegenerative disorders (10). DR development is linked to retinal pigment epithelium (RPE) dysfunction, and modulation of mitophagy may help protect the RPE from various pathological injuries (11), potentially playing a key role in preventing DR onset and progression. However, the specific role of mitophagy in DR progression remains unclear.

As a microvascular complication, DR’s systemic metabolic derangements can be reflected in the expression of blood genes (12). Blood samples offer easy accessibility, repeatable sampling, and convenient dynamic monitoring, making them more suitable for clinical translational applications (12). This study utilized clinical blood transcriptomic data (GSE189005 and GSE221521 datasets) from patients with DR in the GEO database and employed bioinformatics analysis to identify mitophagy-related genes (MRGs) that play a pivotal role in the onset and progression of DR.

## 2 Materials and methods

### 2.1 Data source

**Figure 1:**
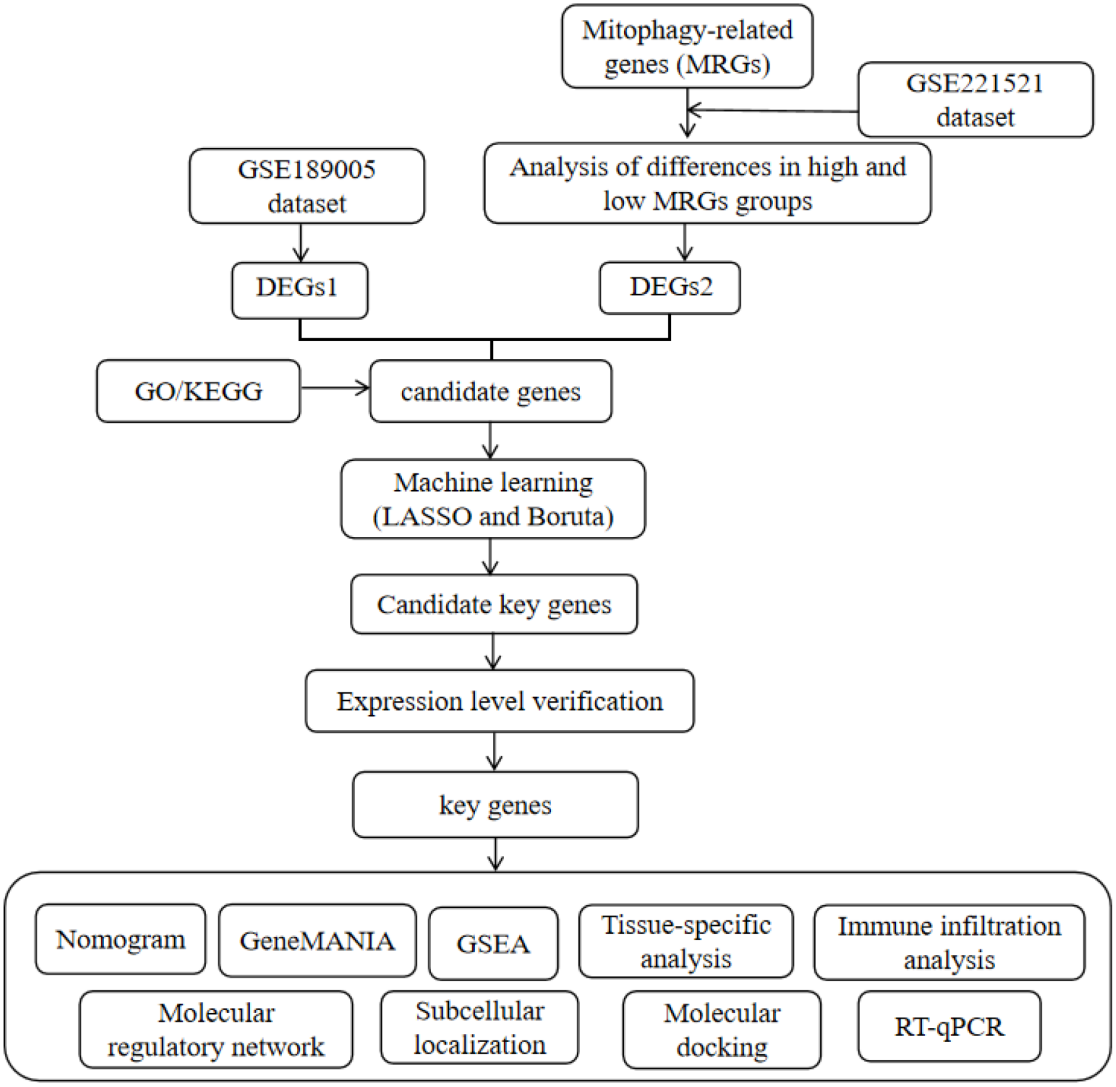
Roadmap of the main research ideas in this article.

The DR-related datasets (GSE189005 and GSE221521) were obtained from the GEO database (http://www.ncbi.nlm.nih.gov/geo/). Specifically, GSE189005 included 10 blood samples from type 2 DM individuals with DR and 9 samples from normal controls, using the GPL570 platform, while GSE221521 (GPL24676) consisted of 69 peripheral blood mononuclear cell samples from patients with DR and 50 from normal controls. GSE99248, derived from the GPL11154 platform, included 16 patients with age-related macular degeneration (AMD) and 15 normal controls, all of whom provided peripheral blood mononuclear cell samples. Additionally, 137 MRGs were extracted from a reference article (13). The analytical workflow for this study is illustrated in **Figure 1**.

### 2.2 Differential expression analysis

In the GSE189005 dataset, differential expression analysis between the DR and control groups was performed using the limma package (v 3.54.0) (14) on expression values normalized by RMA. The thresholds for significance were set at |log_2_FC| > 1 and p.adjust < 0.05, yielding the differentially expressed gene (DEG) set, DEGs 1. Volcano plots and heatmaps of DEGs 1 were generated using ggplot2 (v 3.4.1) (15) and Complex Heatmap (v 2.14.0) (16), respectively. To identify key MRGs for DR, differential expression analysis was performed on the 137 MRGs in the GSE221521 dataset, identifying significantly differentially expressed MRGs with a threshold of p < 0.05. Log_2_(FPKM + 1)-normalized data were used for validation, and a differential violin plot was generated for visualization. Next, in GSE189005, DR samples were classified into high and low MRG score groups based on the median MRG score. Differential expression analysis was conducted using the limma package (v 3.54.0) (18) to identify DEGs 2 between the groups (p-value < 0.05 and |log_2_FC| > 0.5). DEGs 2 were visualized using the same methods as DEGs 1.

### 2.3 Identification of candidate genes

The intersection of DEGs 1 and DEGs 2 was determined, and the overlapping genes were designated as candidate genes. A Venn diagram illustrating this intersection was generated using the Venn Diagram package (v 1.7.1) (17). Subsequently, GO and KEGG enrichment analyses were performed (p < 0.05) (18–20). Additionally, the psych package (v 2.1.6) (21) was used to assess the Spearman’s rank correlation between candidate genes based on their expression levels in DR. Only correlations with an absolute value greater than 0.3 and a significance level of p < 0.05 were included in the construction of a correlation network diagram.

### 2.4 Machine learning

To identify key genes, machine learning analyses using Lasso regression and the Boruta algorithm were performed on the GSE189005 dataset, with DR-affected samples as the dependent variable and the expression levels of candidate genes as the basis for selection. First, Lasso analysis was conducted using the glmnet package (v 4.1-4) (22), identifying candidate key genes 1, whose regression coefficients were not penalized to zero. Subsequently, feature selection was performed using the Boruta package (v 8.0.0) (23), leading to the identification of candidate key genes 2, specifically those confirmed within the analysis. The intersection of candidate key genes 1 and candidate key genes 2 was then used to identify overlapping genes, which were illustrated with a Venn diagram.

### 2.5 Identification of key genes

Next, the expression levels of the intersecting candidate key genes were analyzed in both DR and control samples from GSE189005 and GSE221521. Genes exhibiting consistent expression patterns across both datasets and showing significant differential expression between DR and control samples were designated as key genes. A nomogram model was developed in the GSE189005 training cohort by incorporating the expression levels of these key genes as predictors and DR status as the outcome variable, using the rms package (v 6.5-1) (24). The model’s robustness was validated through AUC analysis and calibration curves. AUC values were calculated using DeLong’s method, while optimal cutoff points were determined by maximizing Youden’s index. Calibration curves were generated through bias-corrected bootstrapping with 1000 replicates. The performance of the nomogram model was further validated in the independent GSE221521 dataset.

### 2.6 Functional enrichment analysis

To identify other genes associated with the function of the key genes, GeneMANIA was employed to predict genes linked to the key genes and their involved processes. Spearman correlation coefficients were calculated for each key gene against all genes in the DR samples from GSE189005, and the correlations were sorted to generate ranked lists. Gene set enrichment analysis (GSEA) was performed using the MSigDB database (http://software.broadinstitute.org/gsea/msigdb), with a threshold of FDR < 0.05. The top 10 enriched pathways were visualized using the enrichplot package (v 1.18.3) (25), with pathways ranked by enrichment score (p-value from smallest to largest).

### 2.7 Tissue-specific analysis

To assess whether the key genes were specifically expressed in the retina, their expression was analyzed using the BioGPS database (http://biogps.org). The retinal tissue expression profiles of these genes were compared to their median expression levels across various tissues and organs. A deviation from the median expression in retinal tissue—whether elevated or reduced—suggested tissue-specific expression for the key genes, potentially identifying them as suitable therapeutic targets for DR.

### 2.8 Immune infiltration analysis

Using the GSE189005 dataset, ssGSEA was applied to analyze immune cell infiltration in the sample gene expression data. A gene set of 28 immune infiltrating cell types was used to calculate the enrichment scores, with the GSVA package (v 1.42.0) (21) employed for computation. Boxplots were generated to visualize the results. The psych package (v 2.1.6) (26) was utilized to analyze the Spearman correlation between the enrichment scores of differentially infiltrating immune cells and key genes (p < 0.05), with a heatmap constructed to illustrate the correlation among the differentially infiltrating immune cells.

### 2.9 Network construction and subcellular localization

To identify transcription factors (TFs) regulating key genes, the JASPAR database (https://jaspar.genereg.net/) was used to predict TFs targeting these genes. The TF-mRNA (key gene) network was visualized using Cytoscape (v 3.8.2) (27). Additionally, miRNAs targeting the key genes were predicted using miRNet (https://www.mirnet.ca/miRNet/home.xhtml) and TargetScan. The predicted miRNAs were further analyzed with miRNet and StarBase databases (http://starbase.sysu.edu.cn/) to identify associated lncRNAs, with the intersection of lncRNAs from both databases used to construct a regulatory network. The ceRNA regulatory network was then visualized using Cytoscape (v 3.8.2) (26). To explore the subcellular localization of key genes and their potential functions, the Genecards database (https://www.genecards.org/) was used to predict the subcellular localization of each gene.

### 2.10 Molecular docking

To identify potential drugs for DR, the DGIdb database (https://dgidb.genome.wustl.edu/) was utilized, and drug-gene interactions were visualized using Cytoscape (v 3.8.2) (26). Molecular docking between key genes and predicted drugs was performed by first obtaining the 3D structures of the drugs and retrieving protein crystal structures from the PDB (https://www.rcsb.org/). Molecular docking was conducted using the CB-Dock database (http://clab.labshare.cn/cb-dock/php/blinddock.php).

### 2.11 RNA extraction and real-time quantitative detection

Five pairs of blood samples were collected from Hebei General Hospital, including five DR samples and five controls. All participants provided informed consent, and ethical approval was granted by Ethical Committee of Hebei General Hospital (No. 2025-LW-0038). The samples were processed for cell isolation and extraction, followed by total RNA extraction using TRIzol reagent (Ambion, USA). RNA quantification was performed using a NanoPhotometer N50 instrument. cDNA synthesis was then carried out using the SweScript First Strand cDNA Synthesis Kit (Servicebio, China) for reverse transcription of mRNA. For RT-qPCR analysis, cDNA was diluted 5–20 times with ddH_2_O. The reaction mixture was prepared as follows: 3µL cDNA, 5µL 2x Universal Blue SYBR Green qPCR Master Mix, and 1µL each of forward and reverse primers (10μM concentration). Primer sequences are provided in **Supplementary Table S1**.

The amplification program included an initial denaturation at 95 °C for 1 minute, followed by 40 cycles consisting of denaturation at 95 °C for 20 seconds, annealing at 55 °C for 20 seconds, and extension at 72 °C for 30 seconds. Data were analyzed using the widely accepted 2^–ΔΔCt^ method, with GAPDH as the internal reference gene for accurate normalization.

### 2.12 Statistical analysis

Statistical analysis was performed using R software (v 4.1.0), and comparisons between groups were conducted using the Wilcoxon test. A p-value of less than 0.05 was considered statistically significant, unless otherwise specified.

## 3 Results

### 3.1 Candidate genes analysis revealed enriched pathways and protein interactions

**Figure 2:**
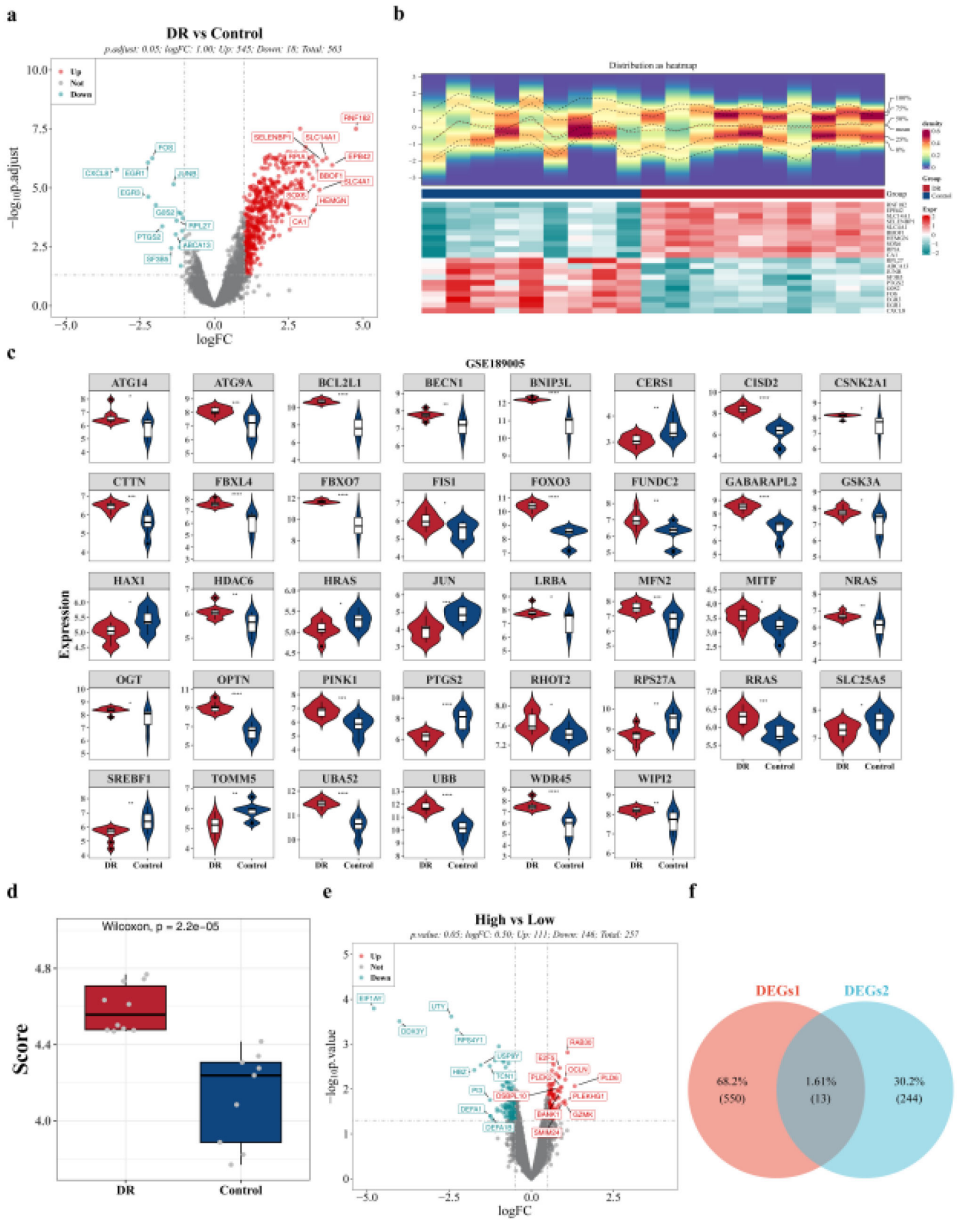
Identification of mitophagy-related candidate genes in DR based on the GSE189005 dataset. **(a)** Volcano plot of DEGs 1 between the DR group and the control group in GSE189005 datase (DR: n = 10; Control: n = 9). Differential analysis was performed using the limma package, and the screening threshold was |log_2_ fold change (log_2_FC)| > 1 and adj.p.Val < 0.05. Red dots represent upregulated genes, green dots represent downregulated genes, and gray dots represent genes with no significant difference. **(b)** Heatmap of the top 20 DEGs 1 sorted in descending order of |log_2_ fold change (log_2_FC)|. **(c)** Differential analysis of MRGs between the DR group and the control group in GSE221521 datase (DR: n= 69; Control: n = 50). **(d)** Difference in MRG scores between the DR group and the control group in GSE189005 datase. **(e)** Volcano plot showing the distribution of DEGs 2 between high and low MRG score groups, and the screening threshold was p < 0.05. Red dots represent upregulated genes, green dots represent downregulated genes, and gray dots represent genes with no significant difference. **(f)** Venn diagram of DEGs 1 and DEGs 2. DEGs, Differentially Expressed Genes; DR, Diabetic Retinopathy; MRGs, Mitochondrial Autophagy-Related Genes.

Based on the DR and control blood samples from the GSE189005 dataset in the GEO database, a total of 563 DEGs 1 were identified between the DR and control groups, comprising 545 upregulated genes and 18 downregulated genes (**Figure 2a-b**). Additionally, 38 MRGs exhibited significant differential expression between the DR and control groups (**Figure 2c**). These genes included ATG14, ATG9A, BCL2L1, BECN1, BNIP3L, CERS1, CISD2, CSNK2A1, CTTN, FBXL4, FBXO7, FIS1, FOXO3, FUNDC2, GABARAPL2, GSK3A, HAX1, HDAC6, HRAS, JUN, LRBA, MFN2, MITF, NRAS, OGT, OPTN, PINK1, PTGS2, RHOT2, RPS27A, RRAS, SLC25A5, SREBF1, TOMM5, UBA52, UBB, WDR45, and WIPI2. A boxplot of these 38 differential MRGs (**Figure 2d**). The DR group exhibited significantly higher MRG scores compared to the control group, highlighting a notable difference that warrants further investigation. Concurrently, the GSE189005 dataset revealed 257 DEGs 2 (high vs. low), with 111 upregulated and 146 downregulated genes (**Figure 2e**). By intersecting DEGs 1 and DEGs 2, 13 overlapping genes were identified: TSPAN5, IFIT1B, PLEK2, PITHD1, MAP3K7CL, HEPACAM2, SLC1A5, ZDHHC2, SMIM24, SLC2A1, MFSD14B, B4GALT3, and RPS21, which were designated as candidate genes (**Figure 2f**).

**Figure 3:**
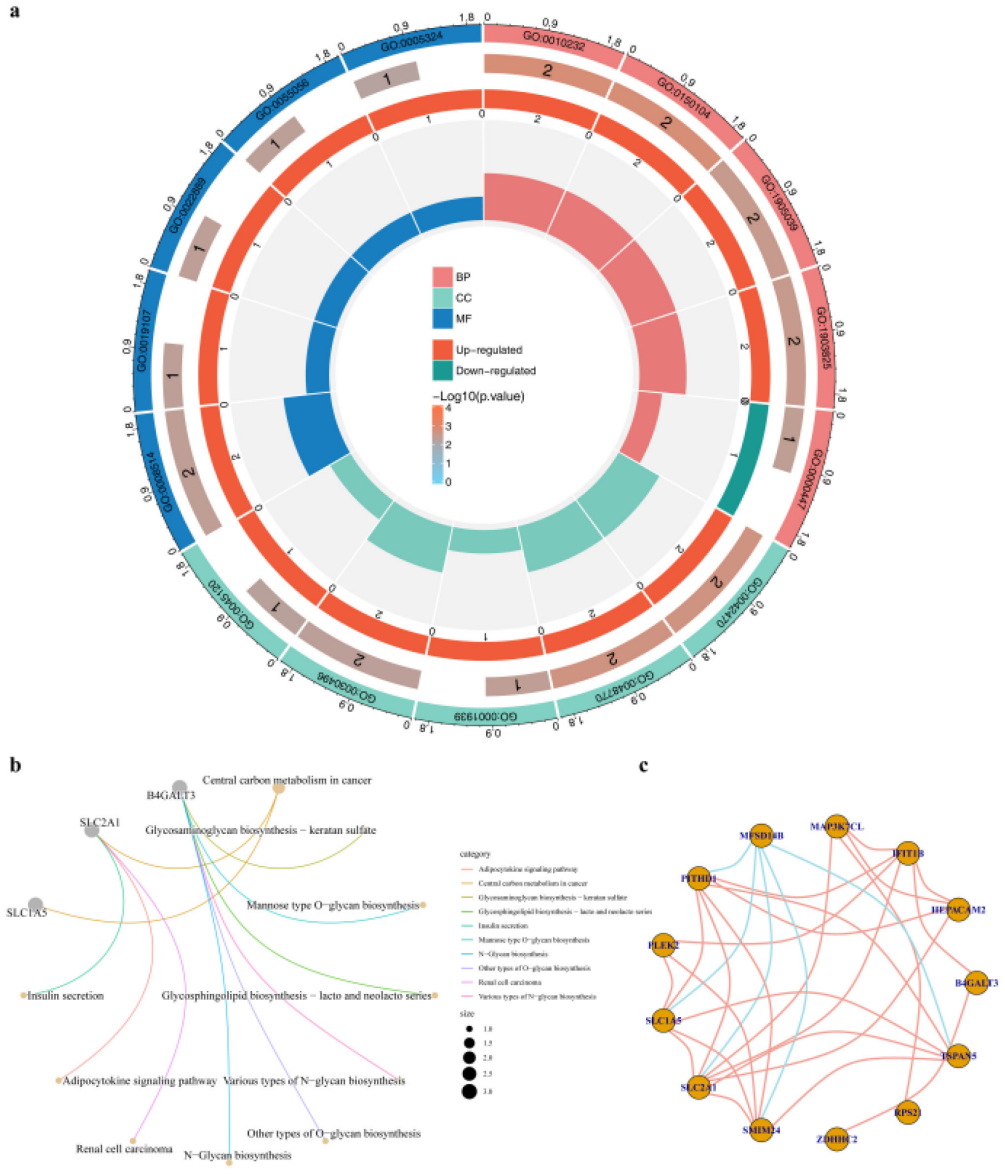
Functional enrichment analysis of candidate genes. **(a)** GO enrichment results of candidate genes. From the outside to the inside, the circles represent GO terms, the number of genes enriched in each GO term, the ratio of upregulated and downregulated genes, and the enrichment factor of each GO term. **(b)** KEGG enrichment results of candidate genes. **(c)** Correlation network diagram of candidate genes. Orange lines represent positive correlations, and blue lines represent negative correlations. GO, Gene Ontology; KEGG, Kyoto Encyclopedia of Genes and Genomes.

GO enrichment analysis revealed that these candidate genes were primarily involved in vascular transport, blood-brain barrier transport, melanosomes, pigment granules, organic anion transmembrane transport activity, and myristoyl transferase activity (**Figure 3a**). KEGG enrichment analysis identified pathways such as glycosaminoglycan biosynthesis of keratan sulfate and ganglioside biosynthesis (lacto and neolacto series) (**Figure 3b**). In the candidate gene correlation network (**Figure 3c**), MFSD14B was notably negatively correlated with multiple genes (p < 0.05).

### 3.2 SLC1A5 and RPS21 were identified as key genes

**Figure 4:**
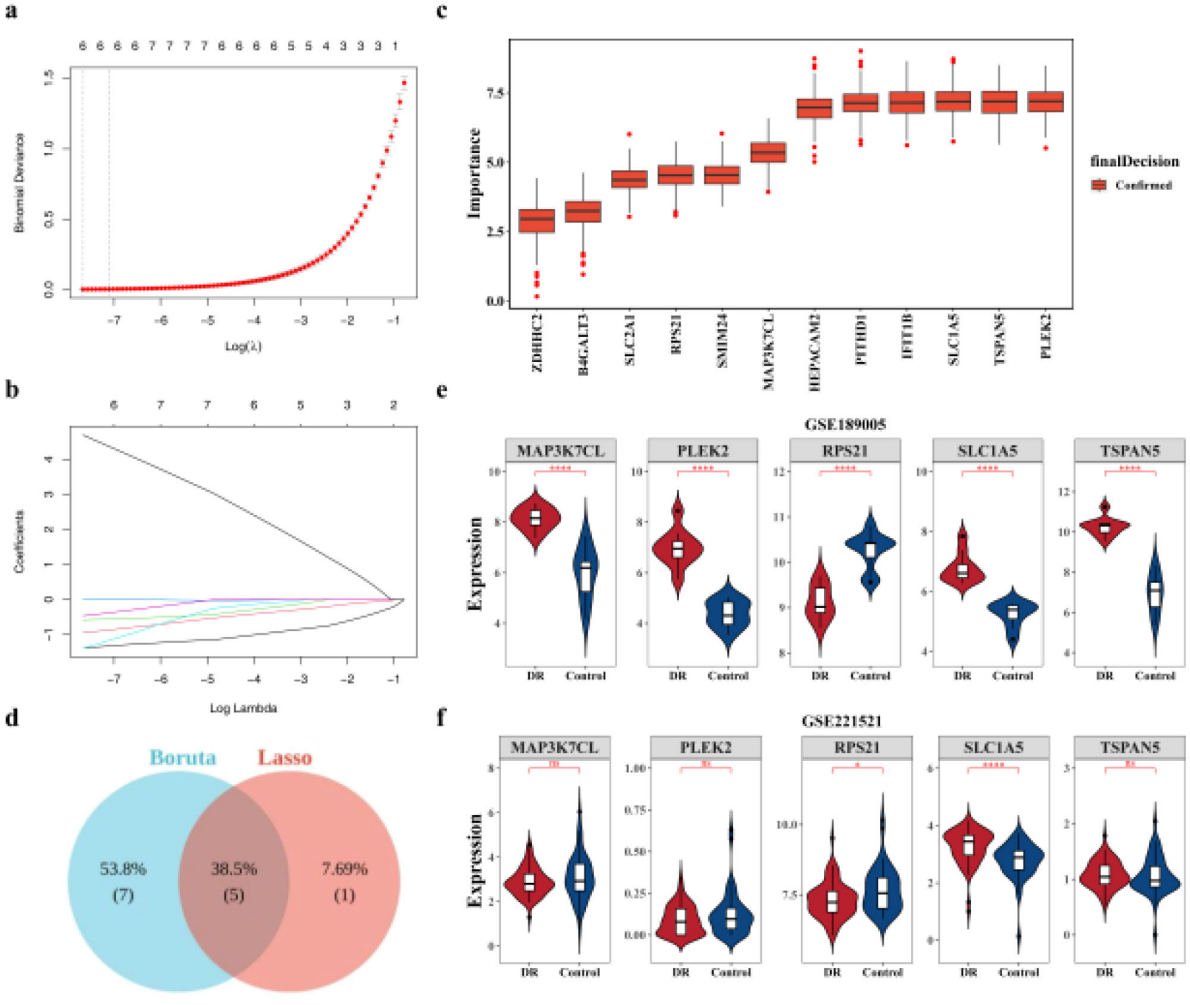
Identification of key genes. **(a, b)** Identification of candidate key genes using the LASSO algorithm. **(a)** Cross-validation for tuning parameters in LASSO analysis. The x-axis represents the logarithm of lambdas, and the y-axis represents the model error. **(b)** LASSO coefficient profile plot. The x-axis represents the logarithm of lambdas, and the y-axis represents variable coefficients. **(c)** Identification of candidate key genes using the Boruta algorithm. The x-axis represents feature genes, and the y-axis represents feature gene importance. The Confirmed module indicates the key gene module. **(d)** Venn diagram of genes obtained by the two algorithms. **(e, f)** Expression level analysis of candidate key genes: **(e)** in the GSE189005 dataset, **(f)** in the GSE221521 dataset. * p < 0.05, ** p < 0.01, *** p < 0.001 were considered statistically significant. LASSO: Least Absolute Shrinkage and Selection Operator.

A machine learning algorithm was applied to screen for candidate key genes from the 13 identified candidate genes. Lasso regression analysis identified six candidate key genes: TSPAN5, PLEK2, MAP3K7CL, SLC1A5, MFSD14B, and RPS21 (**Figure 4a-b**). Simultaneously, the Boruta algorithm identified 12 candidate key genes: TSPAN5, IFIT1B, PLEK2, PITHD1, MAP3K7CL, HEPACAM2, SLC1A5, ZDHHC2, SMIM24, SLC2A1, B4GALT3, and RPS21 (**Figure 4c**). By intersecting the two gene sets, five final intersecting key genes were identified: TSPAN5, PLEK2, MAP3K7CL, SLC1A5, and RPS21 (**Figure 4d**). Notably, SLC1A5 and RPS21 exhibited consistent expression patterns in both GSE189005 and GSE221521 datasets, with significant differential expression between the DR and control groups. These two genes were thus designated as key genes (**Figure 4e-f**). To further evaluate the potential role of SLC1A5 and RPS21 as specific key genes for DR, the GSE99248 dataset was used to examine the expression of these genes in patients with AMD and normal controls. The results showed no significant expression differences between the two groups (**Supplementary Figure S1**).

**Figure 5:**
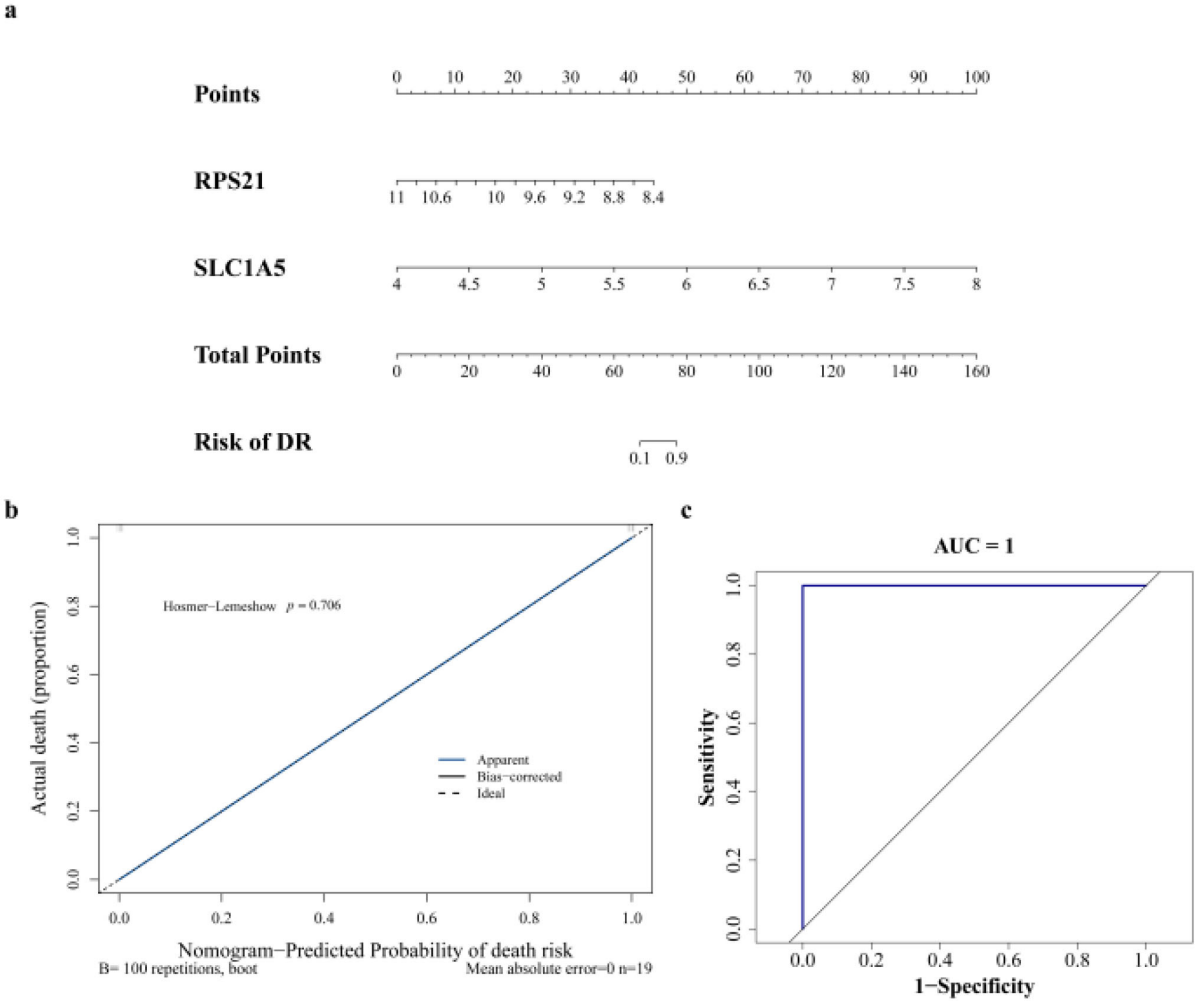
Construction of the nomogram model. **(a)** Construction of a nomogram model based on key genes. The variables in the model are key genes, and “Total Points” represents the sum of individual scores corresponding to the values of all variables. **(b)** Calibration curve of the nomogram model. The x-axis represents the nomogram-predicted probability of disease, and the y-axis represents the actual probability of DR. The black dashed line corresponds to a perfect prediction. The blue solid line represents the actual prediction results, and the black solid line represents the prediction results after bias correction via Bootstrapping (1000 repetitions). **(c)** ROC curve of the nomogram model. The area under the curve enclosed with the coordinate axes is the AUC (Area Under the Curve). ROC, Receiver Operating Characteristic.

Specifically, SLC1A5 was highly expressed in DR, while RPS21 showed lower expression levels within the same group. In the GSE189005 dataset, a nomogram based on these two key genes (SLC1A5 and RPS21) was constructed (**Figure 5a**). The corresponding calibration curve (**Figure 5b**) displayed a Mean Absolute Error (MAE) of less than 0.1, indicating minimal error between actual and predicted DR risk, and demonstrating high precision in the nomogram model. Moreover, the Area Under the Curve (AUC) reached 1.0 (95% CI: 1.000-1.000) (**Figure 5c**), further confirming the model’s strong diagnostic value. The model’s performance was also validated in the independent GSE221521 dataset, which showed favorable predictive accuracy, with an MAE of 0.034, AUC of 0.779 (95% CI: 0.694-0.857), sensitivity of 0.768 (95% CI: 0.649-0.860), and specificity of 0.720 (95% CI: 0.576-0.835) (**Supplementary Figures S2a, b**). These results highlight the robustness and generalizability of the nomogram model based on SLC1A5 and RPS21, suggesting its potential clinical utility for DR risk assessment.

### 3.3 The function analysis of SLC1A5 and RPS21 was helpful for exploring the potential mechanism for DR

**Figure 6:**
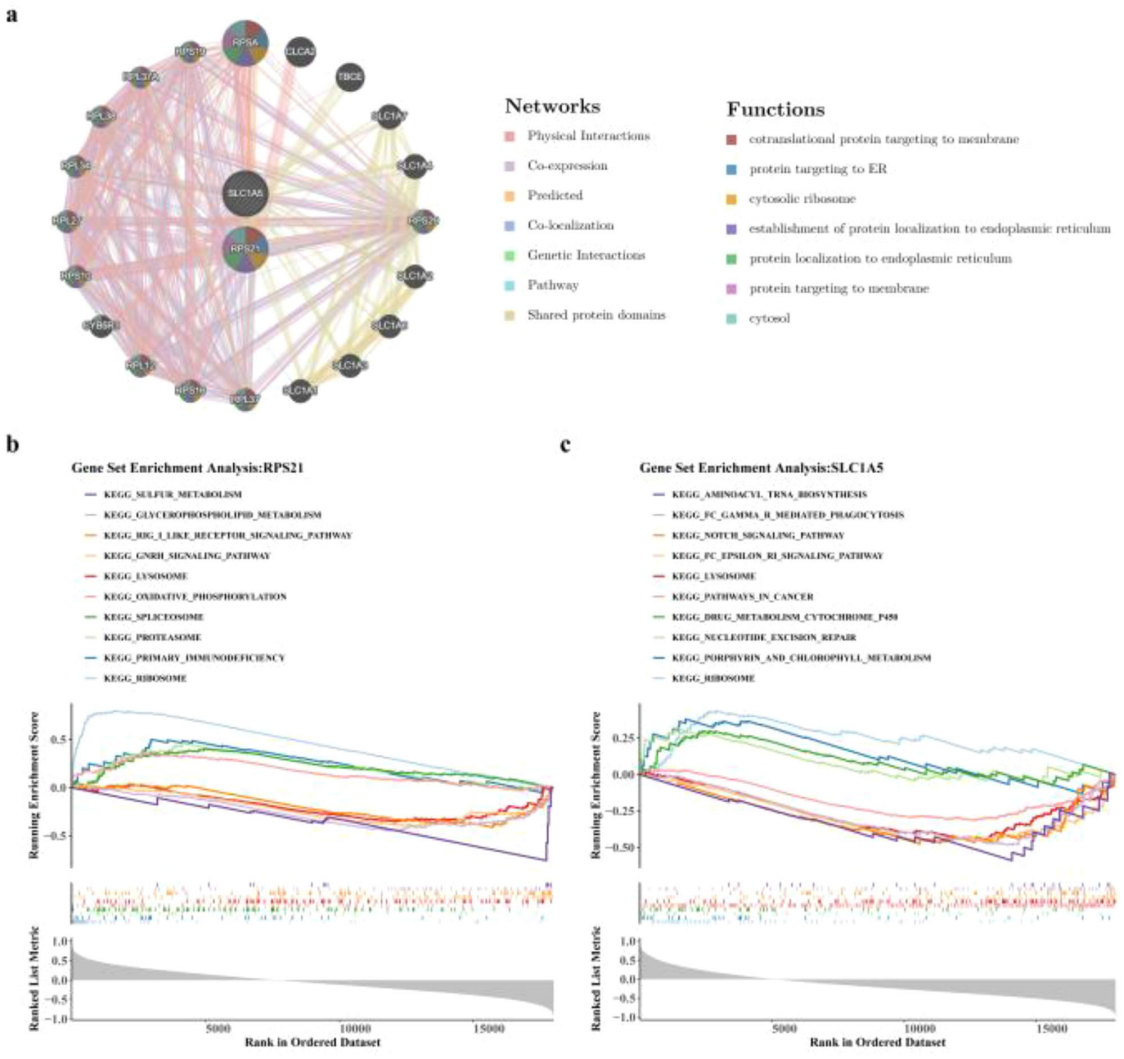
Exploration of key genes. **(a)** GeneMANIA network diagram. The inner circle represents key genes, and the outer circle represents other genes functionally related to the key genes. **(b, c)** GSEA of key genes: **(b)** for RPS21, **(c)** for SLC1A5. The y-axis represents the Enrichment Score (ES). Among them, the ES line chart shows the change trend of the ES score with the accumulation of genes in the ranked gene list. The heatmap shows the correlation coefficients between the ranked key genes and all genes; red usually indicates a positive correlation, and blue indicates a negative correlation. MANIA, Multiple Association Network Integration Algorithm. GSEA, Gene Set Enrichment Analysis.

Gene MANIA analysis identified functionally related genes to SLC1A5 and RPS21, including RPSA, RPS19, RPL37A, and CLCA2 (**Figure 6a**). These genes were primarily involved in processes such as protein targeting to the endoplasmic reticulum (ER), cytosolic ribosome, membrane targeting, and cytosolic functions. Additionally, GSEA enrichment analysis revealed that RPS21 was significantly enriched in 11 signaling pathways (**Figure 6b**), affecting processes like ribosome function, primary immunodeficiency, proteasome activity, spliceosome regulation, oxidative phosphorylation, lysosomal activity, glycerophospholipid metabolism, and sulfur metabolism. In contrast, SLC1A5 was enriched in 21 signaling pathways (**Figure 6c**), predominantly influencing metabolic processes such as ribosome function, porphyrin and chlorophyll metabolism, lysosomal processes, Notch signaling, and aminoacyl-tRNA biosynthesis.

### 3.4 The CD56 dim NK cell and CD56 bright NK cell had a significantly association with key genes SLC1A5 and RPS21

Further analysis revealed that the average expression levels of SLC1A5 and RPS21 across various tissues and organ systems were higher than in retinal tissue (**Table 1**), suggesting their tissue-specific expression.

**Table 1:** Average expression levels of key genes.

Gene	Dataset	ALL tissue	Retina
RPS21	X200834_s_at	3155.55	2601.05
SLC1A5	X208916_at	68.3	65.2

**Figure 7:**
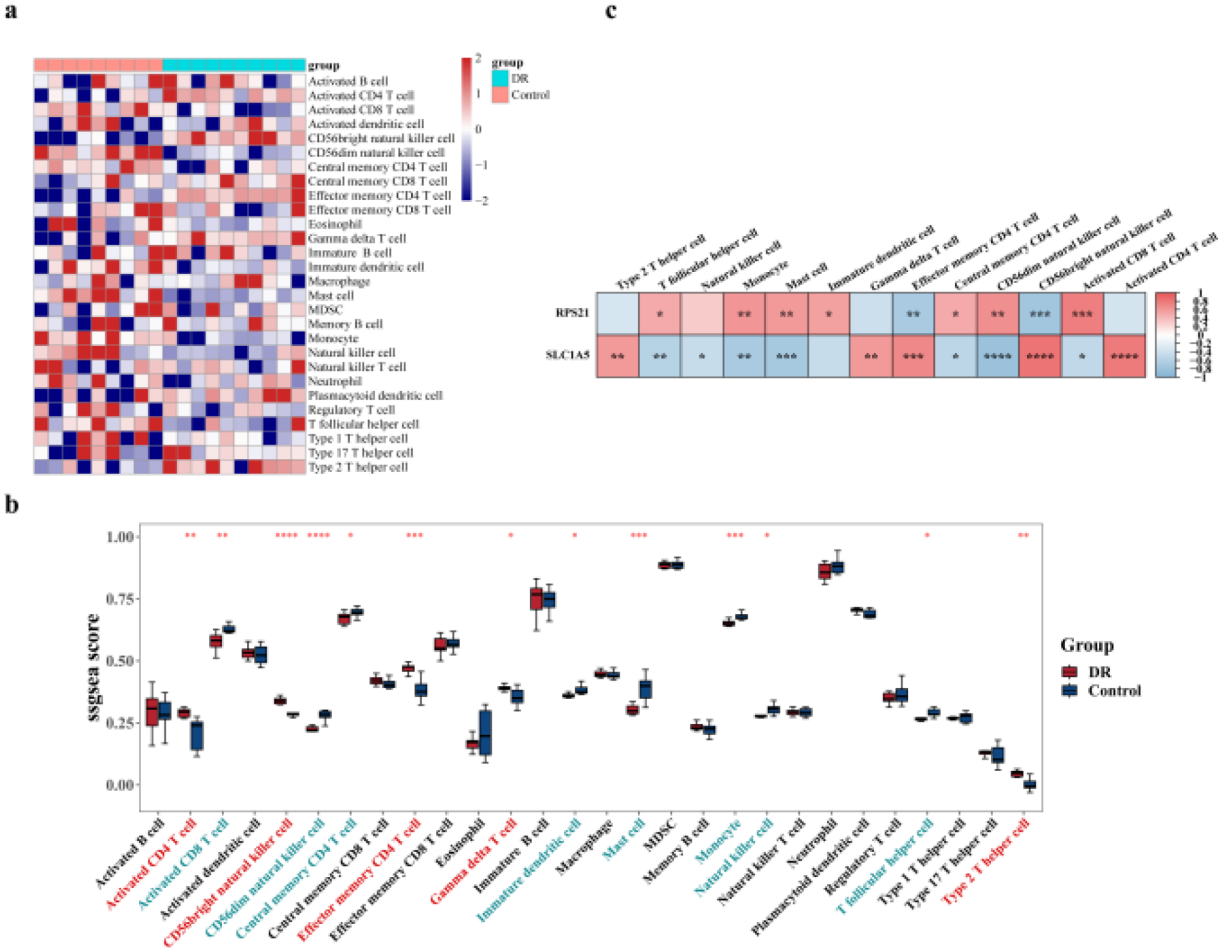
Immune infiltration analysis. **(a)** Immune Infiltration Analysis The enrichment scores of immune infiltrating cells in the DR group and control group from the GSE189005 dataset were calculated based on the ssGSEA algorithm. **(b)** The differences in the abundance of 28 types of immune infiltrating cells between the DR group and control group were compared using the Wilcoxon test. **(c)** The correlation analysis between key genes and differential immune cells was performed. * p < 0.05, ** p < 0.01, *** p < 0.001 were considered statistically significant.

A heatmap visualization (**Figure 7a**) displayed these results. Additionally, significant differences (p < 0.05) were observed in the enrichment scores of 13 types of immune-infiltrating cells in DR (**Figure 7b**). Among these, five immune-infiltrating cell types, including activated CD4 T cells and CD56 bright NK cells, showed significantly higher enrichment scores in DR, while eight types, such as activated CD8 T cells and CD56 dim NK cells, exhibited the opposite trend. Notably, SLC1A5 and RPS21 displayed inverse correlations with these immune cell types (**Figure 7c**). SLC1A5 was most strongly positively correlated with CD56 bright NK cells (r = 0.82) and negatively correlated with CD56 dim NK cells (r = -0.80). RPS21 demonstrated the strongest positive correlation with CD56 dim NK cells (r = 0.77) and the strongest negative correlation with CD56 bright NK cells (r = -0.75).

### 3.5 The regulation network explored the potential mechanism for DR

**Figure 8:**
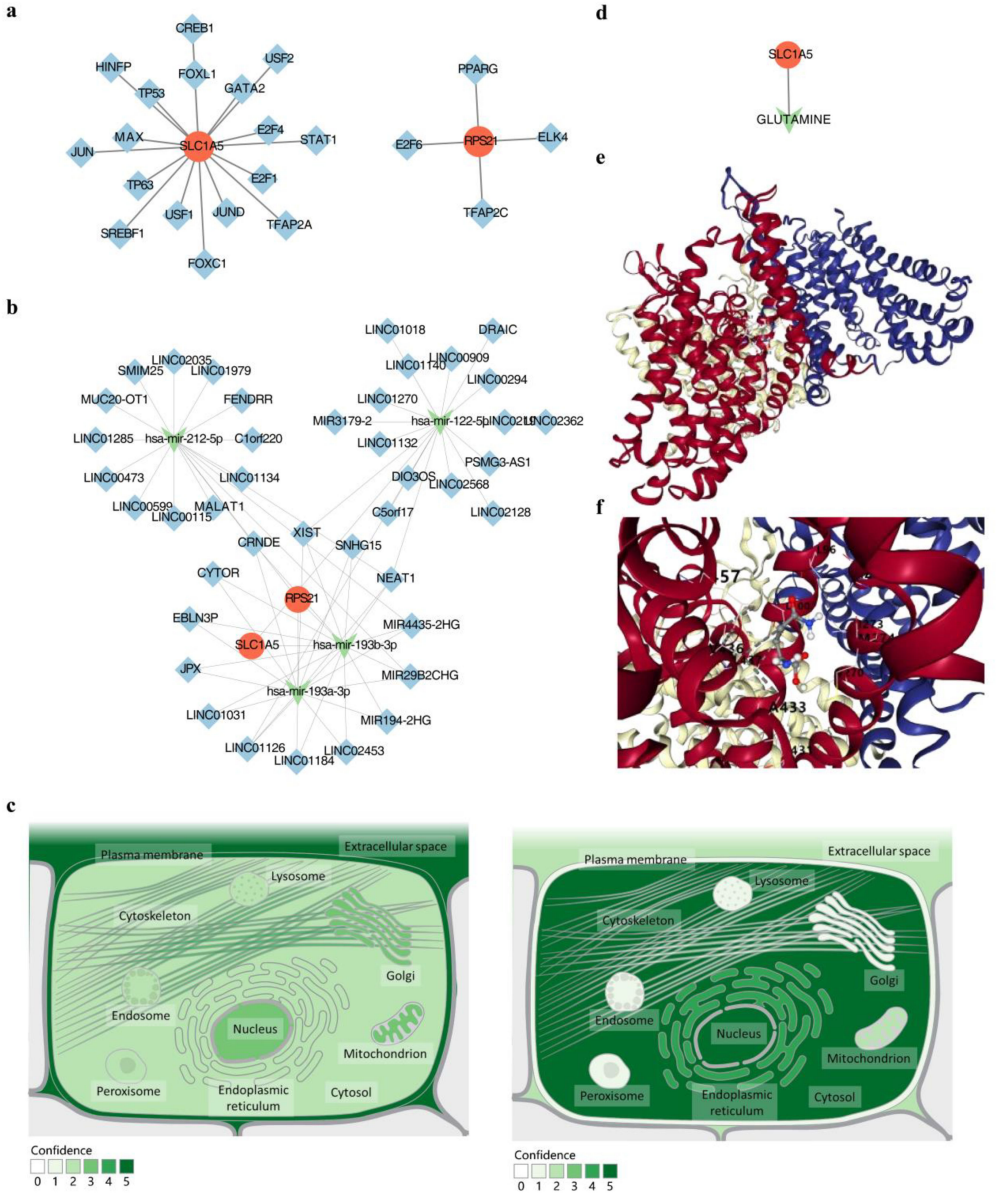
The regulation network explored the potential mechanism for DR. **(a)** TF mRNA regulatory network of key genes. **(b)** ceRNA regulatory network of key genes. **(c)** Key genes of subcellular localization. **(d)** Drug network diagram of key genes. **(e, f)** Docking of SLC1A5 with glutamine molecules. TF: Transcription Factor.

A TF-mRNA (key gene) network was constructed, consisting of 23 nodes (2 key genes and 21 TFs) and 21 edges, integrating the predicted TFs that regulate the key genes (**Figure 8a**). Among these, SLC1A5 had the highest number of predicted TFs (17 TFs, including FOXC1, E2F1, TP53, JUN, etc.), while RPS21 was associated with four predicted TFs (PPARG, E2F6, ELK4, and TFAP2C). In addition, five miRNAs and 41 intersecting lncRNAs were predicted, forming an lncRNA-miRNA-mRNA network with 2 mRNAs, 4 miRNAs, and 41 lncRNAs, totaling 47 nodes and 67 edges (**Figure 8b**). This network illustrated the cooperative regulation of key gene expression by miRNAs and lncRNAs, revealing relationships such as LINC02453-hsa-mir-193a-3p-RPS21 and SNHG15-hsa-mir-193b-3p-SLC1A5. The subcellular localization results showed that SLC1A5 was predominantly localized in the cell nucleus and mitochondria, while RPS21 was mainly found in the cell nucleus, cytoplasm, ER, and mitochondria (**Figure 8c**).

### 3.6 Glutamine could be a potential drug for the treatment of DR

In the search for potential drugs for DR treatment, Glutamine was identified as a predicted drug for SLC1A5 (**Figure 8d**). Further analysis revealed that the binding affinity of SLC1A5 to Glutamine was -4.5 kcal/mol (**Figure 8e-f**), indicating a strong binding capacity between them. This suggests that Glutamine may be a potential therapeutic agent for DR.

### 3.7 RT-qPCR verification

**Figure 9:**
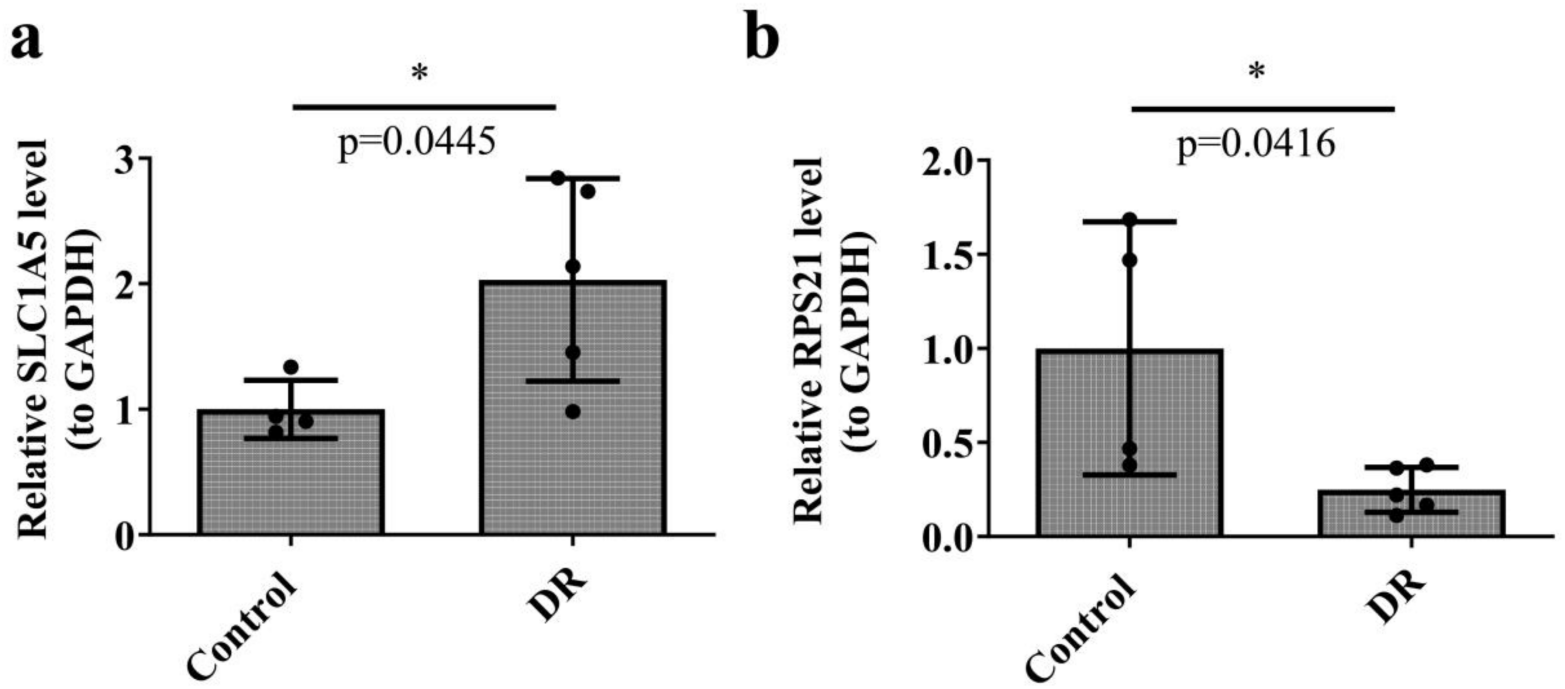
Expression levels of key genes from RT-qPCR analysis. **(a)**. Expression levels of SLC1A5. **(b)** Expression levels of RPS21. Mann-Whitney U test, DR: n = 5; Controls: n = 5, * p < 0.05, ** p < 0.01, *** p < 0.001 were considered statistically significant. RT-qPCR, Reverse Transcription Quantitative Polymerase Chain Reaction.

Additionally, RT-qPCR analysis confirmed the transcriptome analysis results, showing a significant increase in SLC1A5 expression in DR samples, while RPS21 expression was markedly reduced in the same samples (**Figure 9a-b**, **Table 2**).

**Table 2:** The relevant primer sequences are as follows.

Primer	Alignment
SLC1A5 F	TGCAAGCCTAGTTCCAACCC
SLC1A5 R	GGCTGGGTTGGCGTTTTAAG
RPS21 F	TCCGCTAGCAATCGCATCAT
RPS21 R	GCCCCGCAGATAGCATAAGT
Internal reference-GAPDH F	CGAAGGTGGAGTCAACGGATTT
Internal reference-GAPDH R	ATGGGTGGAATCATATTGGAAC

## 4 Discussion

DR, a microvascular complication specific to diabetes, has become a leading cause of blindness (1, 3). Known risk factors include sex, age, diabetes type, family history, disease duration, pregnancy, smoking, and others (28–31). Prolonged hyperglycemia not only damages cone cells and glial cells, disrupting the blood-retinal barrier (32), but also impairs mitochondrial autophagy and cellular function by disrupting the balance between mitochondrial fusion and fission (33). Studies suggest that abnormal mitochondrial autophagy may disrupt mitochondrial function, leading to the accumulation of defective mitochondria and excessive ROS, ultimately causing cellular degeneration and tissue damage (9). Dysregulated mitochondrial autophagy is closely associated with the functional maintenance of the RPE, but its precise regulatory mechanisms in DR remain unclear (10, 11). Therefore, exploring the pathological molecular mechanisms related to mitochondrial autophagy is critical for advancing our understanding of DR pathogenesis and providing a theoretical foundation for therapeutic development. This study utilized data from the GEO public database to compare DR and normal control groups. Through machine learning-based screening and expression level validation, two key genes were identified: SLC1A5 and RPS21. These genes demonstrated robust diagnostic potential in both the training and validation sets. RT-qPCR validation confirmed that their expression patterns were consistent with transcriptomic data, highlighting their likely involvement in DR pathogenesis.

The SLC1A5 gene is located in the nucleus of human cells, encoding a protein that mediates the transport of glutamine from the cytoplasm to the mitochondrial membrane (33). The ammonia produced during glutamine metabolism can stimulate mitochondrial autophagy, regulate lysosomal function, and inhibit mitochondrial autophagy through activation of the mTOR signaling pathway (34). In diabetic kidney disease, a hyperglycemic environment upregulates SLC1A5 expression *via* KPNA2, activating the mTORC1/p70S6K pathway to inhibit autophagy (35). Additionally, p62/SQSTM1 has been shown to directly upregulate SLC1A5 expression (36). Within the p62-SLC1A5-mTORC1 regulatory network, SLC1A5 acts as a key node that coordinates amino acid metabolism, protein synthesis, and oxidative stress responses (37). Furthermore, pathway enrichment analysis in this study indicated that SLC1A5 is significantly involved in metabolic processes, such as ribosome function and aminoacyl-tRNA biosynthesis. In the hyperglycemic state associated with DR, the p62-SLC1A5-mTORC1 regulatory network likely promotes excessive glutamine uptake to sustain mTORC1 signaling activation, inhibiting mitochondrial autophagy. This results in the accumulation of impaired mitochondria, enhances ribosome biogenesis and protein translation, and contributes to metabolic disorders in retinal cells. Concurrently, the altered metabolic patterns driven by this network affect intracellular redox balance. These factors collectively lead to pathological changes, including autophagy dysfunction, abnormal protein accumulation, and heightened oxidative stress (38), ultimately exacerbating neurovascular damage in DR.

This study reports, for the first time, the downregulation of RPS21 in DR, a finding consistent with the significantly reduced levels of RPS21 protein observed in peripheral blood samples from patients with type 1 diabetes mellitus (T1DM) (39). In Fatima et al.’s study, analysis of peripheral blood mononuclear cells from patients with T1DM and multiple sclerosis (MS) revealed RPS21 as a differentially expressed protein involved in nucleotide metabolism and protein synthesis (39). GSEA in the current study showed that RPS21 is primarily enriched in ribosomal pathways, suggesting its potential role in regulating protein translation. Previous studies have demonstrated the upregulation of ribosomal proteins RPL3 and MRPL16 in retinal tissues from patients with DR, with their involvement in disease progression through modulation of cellular metabolism and protein synthesis (40). As a member of the ribosomal protein family, RPS21 may be closely associated with mitochondrial protein homeostasis (41). The downregulation of RPS21 could lead to mitochondrial ribosome dysfunction, impairing the synthesis of mitochondrial-encoded proteins and disrupting mitochondrial quality control. During DR progression, abnormal RPS21 expression may disturb the balance between ribosome and mitochondrial autophagy, resulting in metabolic disorders and retinal cell damage. These findings provide novel insights into the role of ribosomal proteins in diabetic microvascular complications, although the specific mechanisms in DR warrant further investigation.

Through the construction of TF-mRNA and ceRNA regulatory networks for key genes, potential interactions between RPS21, SLC1A5, TFs such as E2F1, and other regulators including XIST and hsa-mir-193b-3p were identified. The study revealed that hsa-mir-193b-3p could specifically inhibit the expression of PPARGC1A/PGC1α in metabolically abnormal states (42). PPARGC1A, a critical regulator of mitochondrial biology and autophagy (43), when downregulated, may lead to mitochondrial dysfunction and impaired autophagy. RPS21 may regulate PPARGC1A expression (42) by interacting with hsa-mir-193b-3p, thereby influencing mitochondrial biogenesis and autophagy, contributing to energy metabolism disorder in retinal cells and playing a role in DR progression. The expression of SLC1A5 may be regulated through dual mechanisms involving lncRNA-XIST and E2F1. XIST may inhibit SLC1A5 by binding to miR-137 (44), enhancing oxidative damage in the retinal ganglion by promoting glutamate transport and ROS production. Conversely, E2F1 may upregulate SLC1A5 expression, enhancing glutamate transport, promoting oxidative stress, and synergizing with the VEGF signaling pathway to exacerbate retinal nerve damage and pathological angiogenesis (44–46). These findings provide new molecular insights into DR pathogenesis and highlight potential targets for RNA interference-based therapeutic strategies.

Considering the pivotal role of immune microenvironment dysregulation in diabetic complications, a systematic analysis of immune cell infiltration patterns in patients with DR was conducted. The results revealed a significant positive correlation between SLC1A5 and CD56-positive natural killer (NK) cells, and a marked positive association between RPS21 and CD56-negative NK cells. In diabetes and its complications, NK cells play a pivotal role in disease progression and exhibit complex biological functions (47). Based on CD56 expression density, NK cells are divided into subgroups with distinct functional characteristics: CD56 bright and CD56 dim (48). In NK cells, SLC1A5 (ASCT2), a key amino acid transporter induced by IL-2, promotes intracellular metabolic reprogramming by enhancing glutamine uptake, leading to continuous activation of the mTORC1 signaling pathway and mitochondrial dysfunction (49). This metabolic disorder may result in mitochondrial damage accumulation due to impaired autophagy and promote excessive production of pro-inflammatory factors like IFN-γ by NK cells, contributing to NK cell dysfunction and fostering an inflammatory microenvironment in DR. The significant positive correlation between RPS21 and CD56 dim NK cells suggests that RPS21 may support ribosome function, ensuring the synthesis of mitochondrial autophagy-related proteins. As the primary effector subpopulation, CD56 dim NK cells may experience mitochondrial autophagy dysfunction, impairing cytotoxicity and weakening immune surveillance against retinal neovascularization. Notably, patients with DR exhibit an imbalance between innate immune activation (e.g., neutrophils) and adaptive immune suppression (e.g., lymphocytes) (50). NK cell dysfunction—characterized by CD56 bright cell hyperactivation and CD56 dim cell functional inhibition—may exacerbate this immune dysregulation, serving as a bridge between innate and adaptive immunity.

This study has several limitations. Firstly, the RT-qPCR validation conducted here differs from the blood sample sources in public databases, which may introduce potential confounding factors. Secondly, although bioinformatics analysis and computational models predicted potential associations between SLC1A5 and RPS21 with mitochondrial autophagy and their possible roles in DR, functional experimental validation is still required. Thirdly, the limited sample size in the dataset may affect the statistical power of the study. Fourth, the interaction between genes and drugs was based solely on bioinformatics analysis and needs experimental verification. Future research should systematically collect peripheral blood and retinal tissue samples from patients at different disease stages to increase sample size. Additionally, larger-scale public databases should be integrated for cross-validation, and multiple *in vitro* experiments, such as gene knockout/expression experiments, single-cell sequencing, and diabetic animal models, should be conducted to further validate SLC1A5 and RPS21 as key diagnostic genes for DR.

## 5 Conclusion

In this study, through bioinformatics analysis and experimental validation, SLC1A5 and RPS21 were identified for the first time as potential diagnostic markers for DR. A nomogram diagnostic model based on these two key genes was also established. The results suggest that these genes may be involved in DR progression by regulating ribosomal function and glutamine metabolism. Furthermore, glutamine may emerge as a potential therapeutic target.

These findings offer novel research directions for the early diagnosis and treatment of DR. However, further experimental validation is essential to confirm these observations.

## Data Availability

The datasets presented in this study can be found in online repositories. The names of the repository/repositories and accession number(s) can be found in the article/**Supplementary Material**.

## Supplementary material

Supplementary Figure 1 The expression of key genes was verified in the GSE99248 dataset (age-related macular degeneration (AMD): n = 16; controls: n = 15). Wilcoxon test, *p < 0.05, **p < 0.01, ***p < 0.001 were considered statistically significant.

Supplementary Figure 2 Validation of the nomogram model in the independent validation set. **(a)** Calibration Curve of nomogram model in GSE221521 dataset. **(b)** ROC curve of nomogram model in GSE221521 dataset.
