# Supplementary material for: Transcriptome analysis and RT-qPCR validation of mitophagy-related key genes in the progression of diabetic retinopathy
Source: Front Endocrinol (Lausanne). 2026 Jan 12;16:1733368. doi: 10.3389/fendo.2025.1733368 (PMC12832545; doi:10.3389/fendo.2025.1733368)
Supplement: Supplementary Figure 1 — The expression of key genes was verified in the GSE99248 dataset (age-related macular degeneration (AMD): n = 16; controls: n = 15). Wilcoxon test, *p < 0.05, **p < 0.01, ***p < 0.001 were considered statistically significant. [file DataSheet1.docx]

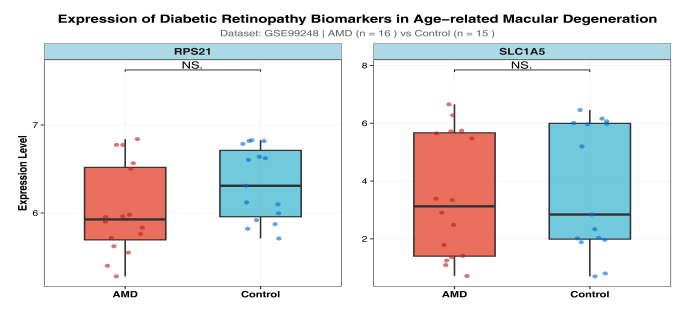


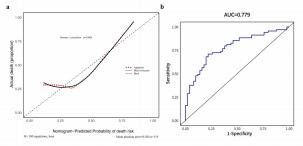


**Supplementary materials**

**Supplementary Fig. S1** The expression of key genes was verified in the GSE99248 dataset (age-related macular degeneration (AMD): n = 16; controls: n = 15). Wilcoxon test, * p < 0.05, ** p < 0.01, *** p < 0.001 were considered statistically significant.

**Supplementary Fig. S2 Validation of the nomogram model in the independent**

**validation set. (a)** Calibration Curve of nomogram model in GSE221521 dataset. **(b)**

ROC curve of nomogram model in GSE221521 dataset.
